# Report of depressive symptoms on waiting list and mortality after liver and kidney transplantation: a prospective cohort study

**DOI:** 10.1186/1471-244X-11-182

**Published:** 2011-11-21

**Authors:** Emmanuelle Corruble, Caroline Barry, Isabelle Varescon, Antoine Durrbach, Didier Samuel, Philippe Lang, Denis Castaing, Bernard Charpentier, Bruno Falissard

**Affiliations:** 1 INSERM U 669, Paris XI University, Psychiatry Department of Bicetre University Hospital, Assistance Publique-Hopitaux de Paris; 94275 Le Kremlin Bicêtre, France; 2 INSERM U 669, Bicetre University Hospital, Assistance Publique-Hopitaux de Paris; 94275 Le Kremlin Bicêtre, France; 3 Paris V University, Hepatology and Surgery department of Paul Brousse University Hospital, France; 4 INSERM U542, Nephrology Department of Bicetre University Hospital, Assistance Publique-Hopitaux de Paris; 5 Head of the hepatology department of Paul Brousse University Hospital, Assistance Publique-Hopitaux de Paris, France; 6 Nephrology Department of Creteil University Hospital, Assistance Publique-Hopitaux de Paris, France; 7 Head of the surgery department of Paul Brousse University Hospital. Assistance Publique-Hopitaux de Paris, France; 8 INSERM U542, Nephrology Department of Bicetre University Hospital, Assistance Publique-Hopitaux de Paris, France; 9 INSERM U669, Paris XI University, Department of Biostatistics and Public Health, Paul Brousse Hospital, Assistance Publique-Hôpitaux de Paris; 94800 Villejuif, France

**Keywords:** depressive symptoms, self-assessment, transplantation, liver, kidney, outcome

## Abstract

**Background:**

Little research has explored pre-transplantation psychological factors as predictors of outcome after liver or kidney transplantation. Our objective is to determine whether report of depressive symptoms on waiting list predicts outcome of liver and kidney transplantation.

**Methods:**

Patients on waiting list for liver or kidney transplantation were classified for report or non-report of depressive symptoms on waiting list. 339 were transplanted 6 months later on average, and followed prospectively. The main outcome measures were graft failure and mortality 18 months post-transplantation.

**Results:**

Among the 339 patients, 51.6% reported depressive symptoms on waiting list, 16.5% had a graft failure and 7.4% died post-transplantation.

Report of depressive symptoms on waiting list predicted a 3 to 4-fold decreased risk of graft failure and mortality 18-months post-transplantation, independently from age, gender, current cigarette smoking, anxiety symptoms, main primary diagnosis, UNOS score, number of comorbid diagnoses and history of transplantation. Data were consistent for liver and kidney transplantations. Other baseline predictive factors were: for graft failure, the main primary diagnosis and a shorter length since this diagnosis, and for mortality, older age, male gender and the main primary diagnosis.

**Conclusion:**

Further studies are needed to understand the underlying mechanisms of the association between report of depressive symptoms on waiting list and decreased risk of graft failure and mortality after transplantation.

## Background

The growing population of patients who have survived liver and kidney transplantation [1,2] has intensified the need to identify risk factors for less favourable outcomes such as graft failure and mortality.

Some risk factors for graft failure and mortality are related to the transplantation and the post-transplantation period. Others are already known for earlier stages, when transplantation candidates are on waiting list. These are recipient characteristics, such as age, gender, diagnosis of primary medical disease, United Network for Organ Sharing (UNOS) priority status, cigarette smoking status or self-reported medication nonadherence and depressive symptoms [3-9].

Whether or not the report of depressive symptoms on waiting list by future recipients predicts liver and kidney transplantation outcomes remains uncertain, since the three prospective studies available for liver [8,10] and for kidney transplantation [4] failed to show significant results, probably because of small sample sizes. Of note, other studies in the field of transplantation showed divergent results: two studies in heart transplantation showed either a poorer outcome in patients with pre-transplantation depressive symptoms [11] or non-significant results [12] whereas a study in lung transplantation [13] showed a better one-year post-transplantation outcome for patients who were depressed before transplantation.

The paucity of literature assessing whether depressive symptom report on waiting list is a risk factor for solid organ transplantation outcome is surprising considering the extraordinary stress associated with organ failure and terminal illness, the complex process involved in selecting transplant candidates for scarce organs, the period of waiting for an available donor transplant, the uncertainty of surviving the surgery and any postoperative complications, and the anticipated complications of immunosuppressant treatment.

Our hypothesis is that a high level of report of depressive symptoms on waiting list predicts poor outcome post-transplantation. Our objective was to determine whether report of depressive symptoms on waiting list for liver or kidney transplantation would be associated with transplantation outcome.

## Methods

### Study design and population

**Figure 1:**
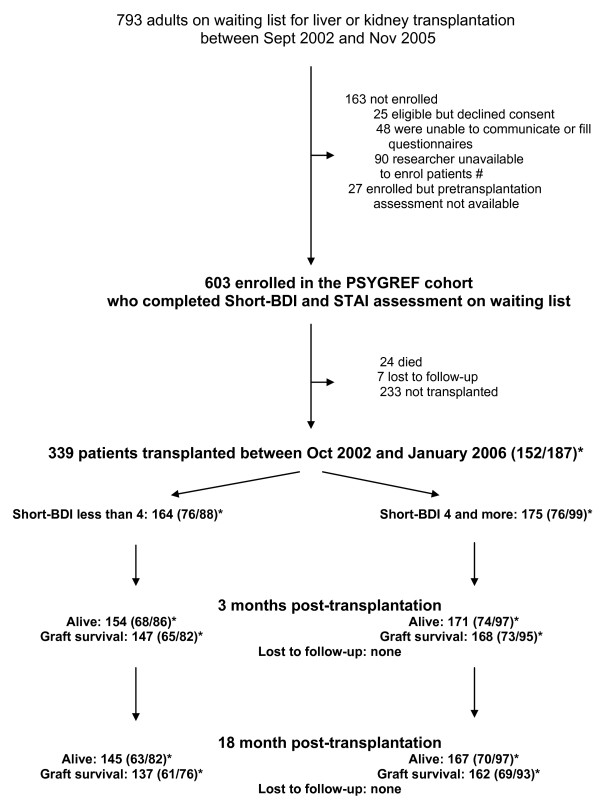
**Flow of participation and number of events**. # Only one half-time researcher per center sought consent for participation and assessed patients. When this researcher was unavailable, new patients could not be enrolled. *Numbers of patients are given for the whole sample and for liver and kidney transplantation into brackets (liver/kidney).

PSYGREF is a prospective longitudinal observational cohort of adult patients. Its main objective is to assess the relationship between psychological variables and outcome of liver and kidney transplantation. Patients were assessed on waiting list from September 1, 2002 in the three kidney and liver transplantation centres in the southern area of Paris, France. The present analysis was conducted on patients transplanted before February 2006 and followed up until July 2007, and focused on report of depressive symptoms (Figure 1).

The PSYGREF procedures were approved by the ethics committee of the University Hospital of Bicetre, the institutional review board of the Clinical Research Department of Paris. Data were used according to the standard regulations of the French network for transplantation and for the preservation of patient anonymity and privacy. On account of ethical considerations and in order to avoid bias arising from additional visits, PSYGREF procedures and assessments were conducted when patients had an appointment at the transplantation centre for usual medical visits.

After inclusion on waiting list in the liver and kidney transplantation centres of Paris-XI and XII universities (Centre Hepato-Biliaire of Paul Brousse Hospital, Nephrology Departments of Bicetre and Mondor Hospitals), the medical investigator offered patients the possibility of entering the PSYGREF cohort. Eligible participants were at least 18 years old and had sufficient French language proficiency to complete the assessments. Patients unable to communicate or complete questionnaires, or referred for an emergency transplantation were excluded. Patients were informed that their psychological results would not be known by the transplantation staff. Each patient provided written informed consent. Psychological assessments were conducted by a trained clinical psychologist, blind to medical data. The medical staff was also blind to psychological data. PSYGREF medical data were collected independently from the psychologist interview. They were validated by the PSYGREF scientific board.

### Number of patients at each step are shown in figure 1

The median length between baseline assessment and transplantation was 28 weeks (interquartile range 10-60).

### Baseline assessment

Because many patients were accessible for only brief periods of time, brief instruments were selected.

The standardized self-administered Beck Depression Inventory - Short form (Short-BDI) [14] was used to assess depressive symptoms. The Short-BDI is a brief 13-item self-report inventory, comprising few somatic items, designed and recommended for assessing report of depressive symptoms in patients with medical illnesses [15]. Each item is scored on a 4-point likert scale from 0 to 3. It takes approximately 3-5 min to complete. Cut-off scores for severity of depressive symptoms are available [16]: none or minimal 0[1-3]; mild [4-7]; moderate [8-15]; severe [16 and above]. The binary variable "Short-BDI score less than 4 (yes/no)" was the main explanatory variable. Report of depressive symptoms was thus defined by a short-BDI score of 4 or more.

The 20-item self-report State Trait Anxiety Inventory (STAI) [17], a self-report measure of current anxiety symptoms, focusing on another specific facet of emotional distress, was used to determine whether anxiety and depressive symptoms are differentially related to transplantation outcomes. Since STAI scores were normally distributed, the raw STAI score was used at the early stage of the univariate analysis as a secondary explanatory variable.

Ongoing mental health care, comprising either current psychological or psychiatric treatment or psychotropic drug treatment, was also recorded as a categorical variable (yes/no).

### Outcome ascertainment

The 339 transplanted patients were assessed 3 and 18 months post-transplantation for outcome variables. None of the 339 patients was lost to follow-up (Figure 1).

The main outcome measure was 18-month graft survival, i.e. absence of graft failure. It was recorded as a categorical variable (yes/no). Graft failure was defined either by death or irreversible graft loss. Irreversible graft loss was defined as retransplantation for liver grafts and return to chronic dialysis for kidney grafts.

The secondary outcome measure was 18-month patient survival, i.e. absence of death. It was recorded as a categorical variable (yes/no). Deaths from all causes were ascertained by active follow-up through transplantation centre, medical centres that referred patients to the transplantation centre and family physicians.

### Data analysis

Statistical analyses were performed using the R 2.4.0 package [18]. All tests were two-tailed.

The analysis strategy was defined a priori, based on the main objective of the analysis, which was to determine whether report of depressive symptoms by patients on waiting list would be associated with transplantation outcome 18-months post-transplantation.

Bivariate and multivariate analyses adjusting for other risk factors were performed. Kaplan-Meier plots were constructed to illustrate the association between baseline characteristics and event-free survival. For the primary analysis, logistic models were preferred to Cox proportional hazards models to avoid the issue of discrepancies between short-term and long-term survival. Usual regression diagnosis procedures were performed. No colinearity was evidenced among covariates. Information on baseline covariates was more than 99% complete. No imputation was performed.

In order to select explanatory variables in logistic models, two series of multivariate models were designed. The first was based on variables evidenced in the literature (age, gender, diagnosis of primary medical disease, UNOS priority status, number of comorbid medical diseases and cigarette smoking). The second was a stepwise logistic regression model, which minimized the Akaike Information Criterion (AIC). This particular method penalizes over-parameterization, variables being retained if the model improves enough to balance the increasing number of parameters. Since results of both models were similar, we chose to show the stepwise modelling strategy.

The following baseline variables were considered for inclusion in the models: age (expressed as age in years/10), gender, education (higher secondary/university: yes/no), married/cohabiting status (yes/no), current leisure activities (yes/no), current professional activity (yes/no), duration since the main primary medical diagnosis (<1, 1-5, 5-10, >10 years), number of comorbid medical diseases, previous transplantation (yes/no), dual transplantation (yes/no), current cigarette smoking (daily/occasional/no), UNOS score (1: emergency transplantation; 2: continuous hospitalization in an acute care bed for at least 5 days; 3: ongoing interactions with health care system without continuous hospitalization; 4: at home and functioning normally), STAI score and Short-BDI score (report of depressive symptoms: short-BDI score of 4 or more). The main primary diagnoses (glomerulopathies, tubulo-interstitial, vascular or other nephropathies for kidney transplantation; non-cholestatic cirrhosis, hepato-cellular carcinoma, metabolic diseases or other liver diseases for liver transplantation) were forced into all models.

The final model assessed the association of report of depressive symptoms with 18-month graft failure in the presence of the selected covariables.

Similar analyses were performed for patient survival, the secondary endpoint.

Regarding depression scores, the same analyses using a Short-BDI cut-off score of 7, corresponding to the higher quartile of Short-BDI scores were also performed.

## Results

### Baseline pre-transplantation characteristics

The 339 transplanted patients were 48 years old on average (sd = 12). 41.3% were females. 20.4% were current daily smokers, 47.8% had a higher secondary or university qualification, 69.9% were married or cohabiting, 25.4% had a current professional activity and 60.8% reported current leisure activities.

The main primary medical diagnoses were: non cholestatic cirrhosis (mainly viral and alcoholic) (38.2%), hepatocellular carcinoma (28.3%), cholestatic cirrhosis (12.5%), metabolic disorders (19.7%), others (1.3%) for the 152 liver transplantations; and primary glomerulopathies (46.5%), tubulo-interstitial (11.2%), vascular (17.6%) and other nephropathies (24.6%) for the 187 kidney transplantations.

Regarding UNOS score at baseline, 20.9% of patients scored 2, 75.5% scored 3 and 3.5% scored 4.

The median Short-BDI score at baseline was 4 (minimum: 0, maximum: 25, interquartile range: 2-7). The Short-BDI score mean (sd) was 4.8(4.1). 175 (51.6%) patients reported depressive symptoms (Short-BDI score of 4 or more), including 106(31,3%) with mild depressive symptoms, 64(18,9%) moderate and 5(1,5%) severe. 164 (48.3%) patients did not report depressive symptoms (Short-BDI score less than 4). The median STAI score was 36. Its mean (sd) was 37.2(10.3). STAI and Short-BDI scores were correlated (Pearson coefficient = 0.6).

As compared to patients who did not report depressive symptoms, patients who did were younger, more frequently females, living alone, daily cigarette smokers, they had a lower educational status, were less frequently working and less frequently had leisure activities (table 1). They were not significantly different regarding the likelihood of being transplanted (57.5% among patients who did not report depressive symptoms vs 54.0% among those who did (p = 0.38)), the transplanted organ (kidney/liver), UNOS score, the main primary diagnoses and length since this diagnosis (table 1). Moreover, patients who did and did not report depressive symptoms at baseline did not differ in terms of ongoing mental health care at some time during the study (respectively 9.9% and 5.7%; chi-squared: p = 0.09).

**Table 1:** Baseline socio-demographic, psychometric and medical characteristics in « report of depressive symptoms » (short-BDI score of 4 or more) and « non-report of depressive symptoms » (short-BDI score less than 4) subgroups at baseline.

Baseline characteristics	Non-report of depressive symptoms (n = 164)	Report of depressive symptoms (n = 175)	Statistics	p
**Socio-demographic**				
Age (years) (m(sd))	48.7 (12.3)	46.0 (11.2)	t (337df) = 2.1	0.03
Female n (%)	56 (34.1%)	84 (48.0%)	Chi2 (1df) = 6.7	0.01
Higher secondary/university education n (%)	88 (53.7%)	74 (42.3%)	Chi2 (1df) = 4.4	0.04
Married/cohabiting n (%)	122 (74.4%)	115 (65.7%)	Chi2 (1df) = 3.0	0.08
Current professional activity n (%)	50 (30.5%)	36 (20.6%)	Chi2 (1df) = 4.4	0.04
Current leisure activities n (%)	117 (71.8%)	88 (50.6%)	Chi2 (1df) = 15.9	0.0007
**Psychometric**				
STAI (m(sd))	31.66 (7.24)	42.38 (10.04)	t (337df) = -11.2	< 10 ^-4^
**Medical data**				
Main primary diagnosis			Chi2 (7df) = 8.72	0.27
Glomerulopathies n (%)	35 (21.3%)	52 (29.7%)		
Tubulointerstitial nephropathies n (%)	8 (4.9%)	13 (7.4%)		
Vascular nephropathies n (%)	18 (11.0%)	15 (8.6%)		
Other nephropathies n (%)	27 (16.5%)	19 (10.9%)		
Non cholestatic cirrhosis n (%)	24 (14.6%)	34 (19.4%)		
Hepatocellular carcinoma n (%)	24 (14.6%)	19 (10.9%)		
Metabolic diseases n (%)	17 (10.4%)	13 (7.4%)		
Other liver diseases n (%)	11 (6.7%)	10 (5.7%)		
Length since the main primary diagnosis			Chi2 (3df) = 2.0	0.56
<1 year n (%)	10 (6.1%)	18 (10.3%)		
1 to 5 years n (%)	58 (35.4%)	59 (33.9%)		
5 to 10 years n (%)	38 (23.2%)	37 (21.3%)		
>10 years n (%)	58 (35.4%)	60 (34.5%)		
UNOS score			Chi2 (2df) = 5.4	0.07
2 n (%)	41 (25.0%)	30 (17.1%)		
3 n (%)	120 (73.2%)	136 (77.7%)		
4 n (%)	3 (1.8%)	9 (5.1%)		
Current cigarette smoking			Chi2 (2df) = 13.05	0.001
No n (%)	134 (81.7%)	117 (66.9%)		
Occasional n (%)	10 (6.1%)	9 (5.1%)		
Daily n (%)	20 (12.2%)	49 (28.0%)		

Patients were classified into two groups:

"Non-report of depressive symptoms" for those with a short-BDI score less than 4,

"Report of depressive symptoms" for those with a short-BDI score of 4 or more.

STAI: State Trait Anxiety Inventory

### Transplantation characteristics

Comparisons of the 339 transplanted patients on the transplantation characteristics showed no significant differences between individuals who did and those who did not report depressive symptoms at baseline: number of weeks between baseline assessment and transplantation (Wilcoxon: p = 0.68), number of years of dialysis for kidney transplantation (median: 3 years among patients who did report depressive symptoms vs 2.5 years among those who did not, Mann&Whitney W = 4322, p-value = 0.92), frequency of living donors (19.4% among patients who did report depressive symptoms vs 19.5% among those who did, chi-squared: p = 0.98), frequency of surgical repairs (16.8% among patients who did report depressive symptoms vs 19.5% among those who did not, chi-squared: p = 0.51), median length of hospitalization for transplantation (25 days among patients who did report depressive symptoms vs 24 days among those who did not, Wilcoxon: p = 0.39), and number of immuno-suppressants recorded at time of discharge from hospital following transplantation (chi-squared: p = 0.66).

### Outcomes

Graft and patient survival rates (Table 2) were obtained for all transplanted patients. Mortality was due to infection (n = 10), acute liver failure (n = 5), recurrence of the initial disease (n = 2), cardio-vascular causes (n = 2) for liver transplantation, and infection (n = 5), graft rejection (n = 1) and cardio-vascular causes (n = 2) for kidney transplantation.

**Table 2:** Rates of graft failure and all-cause mortality, in patients who did and did not report depressive symptoms at baseline.

	Non-report of depressive symptoms (n = 164)			Report of depressive symptoms (n = 175)		
	All	Liver (n = 76)	Kidney (n = 88)	All	Liver (n = 76)	Kidney (n = 99)
18-month graft failure %	16.5%	19.7%	13.6%	7.4%	9.2%	6.1%
18-month all-cause mortality %	11.6%	17.1%	6.8%	4.6%	7.9%	2%

### Bivariate analyses

**Figure 2:**
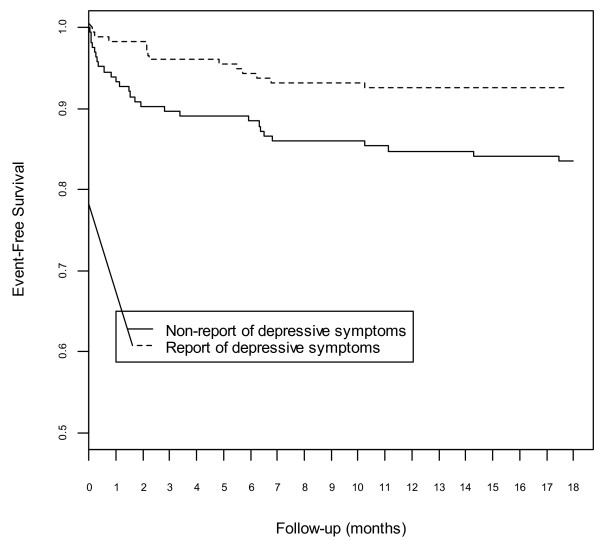
**Cumulative incidence of graft survival based on report of depressive symptoms on waiting list**. Patients were classified into two groups: "Non-report of depressive symptoms" for those with a short-BDI score less than 4. "Report of depressive symptoms" for those with a short-BDI score of 4 or more.

Patients who did report depressive symptoms at baseline had lower rates of graft failure and mortality than those who did not (table 2). Figure 2 shows Kaplan-Meier curves for 18-month graft survival according to report of depressive symptoms at baseline.

7.4% of patients had graft failure among individuals who did report depressive symptoms at baseline compared to 16.5% among those who did not (OR, 0.41; 95%CI, 0.20-0.82; p = 0.01) (table 3). The other baseline characteristics were not significantly associated with 18-month graft failure.

**Table 3:** Univariate and multivariate effects of "report of depressive symptoms" at baseline on 18-month outcomes.

	Odds Ratio (95% CI)*	p
**Non-adjusted univariate analysis**		
Graft failure	0.41 [0.20; 0.82]	0.01
Mortality	0.37 [0.16; 0.88]	0.02
Analysis adjusted for liver/kidney transplantation		
Graft failure	0.40 [0.20; 0.83]	0.01
Mortality	0.37 [0.16; 0.88]	0.02
Multivariate stepwise logistic regression		
Graft failure §	0.37 [0.17; 0.78]	0.01
Mortality §§	0.25 [0.08; 0.83]	0.02

* Odds ratio for comparison with the « non-report of depressive symptoms» subgroup.

CI indicates confidence interval.

§ Adjustment for: age, gender, main primary diagnosis and length since this diagnosis.

§§ Adjustment for: age, gender, main primary diagnosis, length since this diagnosis, history of transplantation and STAI score.

4.6% of patients died among those who did report depressive symptoms at baseline compared to 11.6% among those who did not (OR, 0.37; 95%CI, 0.16-0.88; p = 0.02) (table 3). Older age (p = 0.02) and male gender (p < 0.01) were also significantly associated with 18-month mortality.

No significant effect of depressive symptom intensity was shown either on 18-month graft survival or patient survival.

Eleven of the thirteen Short-BDI items contributed to results obtained on its total score (sadness OR 0.2, dissatisfaction OR 0.36, self-image change OR 0.45, social withdrawal OR 0.49, anorexia OR 0.54, self-dislike OR 0.59, pessimism OR 0.68, work difficulty OR 0.85, sense of failure OR 0.87, guilt OR 0.93, self-harm OR 0.94, fatigability OR 1.11 and indecisiveness OR 1.28), ruling out the hypothesis that the association between graft survival and report of depressive symptoms might be related to specific individual items.

Using a Short-BDI cut-off score of 7 corresponding to the highest quartile of Short-BDI scores, the following results were shown. 6.67% of patients (n = 6) had a graft failure among the 90 individuals with a Short-BDI score higher than 7 at baseline, compared to 13.65% (n = 34) among the 249 others (OR, 0.45; 95%CI, 0.18 -1.12; p = 0.08). 3.33% of patients (n = 3) died among individuals with a Short-BDI score higher than 7 at baseline, compared to 9.64% (n = 24) among the others (OR, 0.32; 95%CI, 0.09-1.1; p = 0.07).

Baseline STAI scores were not associated with graft failure (OR, 0.98; 95%CI, 0.94-1.01; p = 0.15) or mortality (OR, 0.99; 95%CI, 0.95-1.03; p = 0.50).

### Multivariate analyses

Logistic regressions adjusted for liver/kidney transplantation also showed that individuals who did report depressive symptoms at baseline had lower rates of graft failure than those who did not (table 3). Results were consistent in the kidney subgroup (OR, 0.30; 95%CI, 0.09-1.43; p = 0.09) and the liver subgroup (OR, 0.42; 95%CI, 0.15-1.16; p = 0.07).

Logistic regressions adjusted for liver/kidney transplantation evidenced similar results for all-cause mortality (table 3).

After adjusting for confounding parameters using a stepwise logistic regression, the association between report of depressive symptoms and transplantation outcome remained significant: 18-month graft failure was independently predicted not only by the report of depressive symptoms on waiting list, but also by the main primary diagnosis and a shorter length since this diagnosis (tables 3 and 4); 18-month post-transplantation mortality was independently predicted not only by the report of depressive symptoms on waiting list, but also by the main primary diagnosis, older age and male gender (tables 3 and 5). Other variables did not significantly predict graft failure or mortality (table 4 and 5).

**Table 4:** Multivariate model predicting 18-month graft failure.

Baseline Predictor	Odds Ratio	Coefficient (95% CI)	P value	P value for overall test
Report of depressive symptoms (versus non-report)	0.37	[0.17; 0.78]	0.01	
Male (vs female)	1.98	[0.91; 4.30]	0.08	
Age (10 years more)	1.39	[1.00; 1.93]	0.05	
Main primary diagnosis				0.04§
Non cholestatic cirrhosis	1.98	[0.67; 5.89]	0.22	
Hepatocellular carcinoma	0.46	[0.14; 1.45]	0.19	
Metabolic disorders	1.73	[0.58; 5.19]	0.33	
Others liver diseases	2.67	[1.24; 5.72]	0.01	
Glomerulopathies	1.21	[0.58; 2.54]	0.61	
Tubulo-interstitial nephropathies	1.37	[0.41; 4.61]	0.61	
Vascular nephropathies	0.33	[0.08; 1.28]	0.11	
Others nephropathies	0.44	[0.14; 1.38]	0.16	
Length since the main primary diagnosis	*	*		0.005§
<1 year	1.41	[0.61; 3.28]	0.43	
1 to 5 years	0.34	[0.17; 0.70]	0.003	
5 to 10 years	1.12	[0.57; 2.22]	0.74	
>10 years	1.86	[1.03; 3.37]	0.04	

OR, Odds Ratio for 18-month graft failure

CI, Confidence Interval

**Table 5:** Multivariate model predicting 18-month mortality.

Baseline Predictor	Odds Ratio	Coefficient (95% CI)	P value	P value for overall test
Report of depressive symptoms (versus non-report)	0.25	[0.08 ; 0.83]	0.02	
STAI	1.05	[0.99 ; 1.11]	0.14	
Male (vs female)	9.03	[2.38 ; 34.22]	0.001	
Age (10 years more)	1.91	[1.22 ; 3.01]	0.005	
Main primary diagnosis				0.01 §
Non cholestatic cirrhosis	3.07	[0.81 ; 11.66]	0.10	
Hepatocellular carcinoma	0.54	[0.15 ; 1.95]	0.35	
Metabolic disorders	2.42	[0.63 ; 9.23]	0.20	
Others liver diseases	4.98	[1.87 ; 13.25]	0.001	
Glomerulopathies	0.58	[0.18 ; 1.82]	0.35	
Tubulo-interstitial nephropathies	0.74	[0.09 ; 5.98]	0.78	
Vascular nephropathies	0.23	[0.03 ; 1.57]	0.13	
Others nephropathies	0.52	[0.12 ; 2.15]	0.36	
Length since the main primary diagnosis	*	*		0.09 §
<1 year	1.14	[0.38 ; 3.45]	0.81	
1 to 5 years	0.36	[0.15 ; 0.87]	0.02	
5 to 10 years	1.77	[0.75 ; 4.18]	0.19	
>10 years	1.38	[0.55 ; 3.47]	0.50	
History of transplantation	2.98	[0.73 ; 12.15]	0.13	

OR, Odds Ratio for 18-month mortality

CI, Confidence Interval

Using a Short-BDI cut-off score of 7 corresponding to the higher quartile, after adjusting for confounding parameters using multiple logistic regressions, the association between depressive symptoms and 18-month transplantation outcome showed similar odds ratios and remained almost significant (graft failure: OR, 0.42; 95%CI, 0.16-1.09; p = 0.07; mortality: OR, 0.22; 95%CI, 0.04-1.06; p = 0.06).

Results of multivariate analyses for 3-month graft failure and mortality were in line with those for 18-month failure, although not always significant because of the smaller number of events recorded at this time (Figure 1).

## Discussion

About one patient out of two of the cohort reports depressive symptoms on waiting-list for kidney or liver transplantation. These symptoms were mainly of mild intensity, ant to a lesser extent, of moderate intensity. Although lower from those reported in general population, this result is coherent with those of the literature about report of depressive symptoms on waiting list for kidney [4,19,20] or liver [21-25] transplantation. In a context of knowledge pertaining to organ scarcity and waiting list demand, social desirability might lead transplant candidates to under-report the depressive symptoms they are experiencing in order to present themselves as better candidates for transplantation [26]. The specificity of our results regarding report of depressive symptoms as compared to anxiety symptoms suggests that anxiety, but not depressive symptoms may be acceptable from a patient and society point of view in the context of waiting for a solid organ transplantation.

This study shows that report of depressive symptoms on waiting list predicted a 3 to 4-fold decreased risk of graft failure and mortality 18-months post-transplantation. This risk factor is independent from other risk factors such as age, gender, main primary diagnosis and length since this diagnosis. Of note, the risk of death is 3 to 4 times lower for patients who report depressive symptoms on waiting list, suggesting the clinical relevance of this association. Furthermore, data are consistent for liver and kidney transplantations despite differences between these two subgroups for socio-demographic and medical factors. Moreover, using a Short-BDI cut-off score of 7, corresponding to the higher quartile, the association between depressive symptoms and 18-month transplantation outcome showed similar odds ratios and remained almost significant despite small sample sizes. Thus, this result suggests a more general association.

This study is the first prospective cohort study in the field of liver and kidney transplantation showing an association between report of depressive symptoms on waiting list and post-transplantation outcome, since the three previous prospective studies [4,8,9] in this field failed to show significant associations.

The four other prospective studies in the field of solid organ transplantation showed divergent results. One study [12] in heart transplantation was non-conclusive. Another one [11] in heart transplantation also based on self-report of depression showed contradictory results as compared to ours. However, it was conducted in a small subgroup of 57 patients with a specific cardiopathy. And the third one [13] in lung transplantation showed results similar to ours, i.e. a better one-year post-transplantation outcome for patients who had a psychiatric history of depression before transplantation. Recently, our study [9] showed that depressive symptoms 3 months post-liver transplantation and an increase in depressive symptoms between the waiting list and post-liver transplantation periods are associated with an increased risk of long-term mortality. The results of the present study, which show that report of depressive symptoms on waiting-list predicted a 3 to 4-fold decreased risk of graft failure and mortality 18-months post-transplantation, are somewhat different, but compatible with the previous ones. Indeed, the depression score increase between pre and post-transplantation is favored by low pre-transplantation scores. Moreover, the impact of social desirability could explain this difference: whereas social desirability is high in waiting-list, explaining low depression scores and the present association, social desirability is not relevant anymore in the post-transplantation period.

The association of depression with medical outcome has been studied in other fields than transplantation, especially cardio-vascular diseases. Even if almost half of the 57 studies reviewed by Wulsin et al (1999) [27] failed to show any association between depressive symptoms and mortality, several published studies showed that major depression is associated with poorer outcome of medical disorders. Our results are at odds with this literature, which however is controversial, since it failed to show that treating major depression can improve outcome of medical disorders, especially cardio-vascular diseases [28]. Three major points may explain this discrepancy. First, a publication bias may exist, penalising results similar to ours. Secondly, we focused on report of depressive symptoms and not on major depressive episodes as evaluated by clinicians with psychiatric interviews, which are assessed in a large number of published studies. Last but not least, in most studies showing an association between depression and poorer outcome, depressive symptoms were assessed during or just after an acute medical episode [29,30]. In contrast, our study and the Woodman transplantation study [13] assessed depressive symptoms very early in the process of transplantation, i.e. at the beginning of the waiting list period, in the specific context of transplantation candidacy involving social desirability. Yet those other studies are of heart attack, which are indeed acute episodes. Emotional response in the case of those waiting for transplants is a very different case, where there is not an acute episode but a long trajectory of increasingly severe illness and the prospect of death without a transplant.

There is scope for generalising the results of this study on the basis of its main strengths. First, we were able to trace, 18 months post-transplantation, all transplanted subjects from a fairly large cohort of 339 patients who were not medically selected for health status at the time of initial assessment. In addition, many of our results are in line with the literature, not only in term of report of depressive symptoms [21-24], but also in terms of post-transplantation patient and graft survival [2,6,24,31-35], causes of death [2,31,32,34,36] and predictive factors of transplantation outcome [3-7]. Moreover, the major strength of this study is that the assessment of depressive symptoms took place not a few days before transplantation, but 6 months earlier on average. This is specific to this study as compared to other available studies in the field of transplantation [4,10-13].

Nevertheless, the present study has some limitations. We failed to show a relationship between the severity of depressive symptoms reported on waiting list and transplantation outcome. Any correlation would have argued for a causal relationship between these two variables. Importantly, the results of the present study do not address the risks associated with clinical depression but focus on the risk associated with self-report of depressive symptoms. Furthermore, our sample, recruited in 3 transplantation centers, may not be representative of all patients on waiting list for liver or kidney transplantation. And it cannot be ruled out that they may be explained by residual confounding variables, such as non-measured medical characteristics for example.

The mechanisms by which our main result could be explained require further studies. An hypothesis could be that recipients experiencing depressive symptoms on waiting list may be better able to identify and face later psychological difficulties, and thus be better prepared to cope with the significant stressors that occur post-transplantation [13]. Another relevant hypothesis could be that report of lack of depressive symptoms on waiting list may be associated with report of medication non-adherence on waiting list, which has been shown to be associated with a poorer prognosis of transplantation [8]. The role of denial might also be relevant: those who do not acknowledge depression might also be more likely to deny physical symptoms and therefore not seek help when needed or adhere to medications.

## Conclusion

In summary, our results show that patients who report depressive symptoms on waiting list several months before transplantation have a three-fold decreased risk of graft failure and mortality 18-months after kidney or liver transplantation. This risk factor is independent from other established demographic and medical risk factors. Further studies are needed to replicate this result and assess its underlying mechanisms.

## Competing interests

The authors declare that they have no competing interests.

## Authors' contributions

EC, as principal investigator, had full access to all of the data in the study and takes responsibility for the integrity of the data and the accuracy of the data analysis. All authors (EC, CB, IV, AD, DS, PL, BC, DC, BF) read and approved the final manuscript.Study concept and design: EC, BF. Acquisition of data: IV, PL, AD, BC, DS, DC. Analysis and interpretation of data: CB, BF, EC. Drafting of the manuscript: EC, CB, BF.Critical revision of the manuscript for important intellectual content: PL, AD, BC, DS, DC. Administrative, and technical support: EC, BF. Statistical expertise: BF. Obtained funding: EC. Study supervision: EC, BF, BC, DC, PL.

## Funding

Supported by grants from the National Hospital Clinical Research Program of the French Ministry of Health (PHRC AOM 01004) and the Clinical Research Department of the Assistance Publique - Hopitaux de Paris (FAP06011).

Funding organizations had no role in the design or conduct of the study; data collection, analysis, or data interpretation; or preparation, review or approval of the manuscript.

## Pre-publication history

The pre-publication history for this paper can be accessed here:

http://www.biomedcentral.com/1471-244X/11/182/prepub
